# Sucrose treatment of mung bean seeds results in increased vitamin C, total phenolics, and antioxidant activity in mung bean sprouts

**DOI:** 10.1002/fsn3.1269

**Published:** 2019-11-14

**Authors:** Yingying Wei, Xingxing Wang, Xingfeng Shao, Feng Xu, Hongfei Wang

**Affiliations:** ^1^ College of Food and Pharmaceutical Sciences Ningbo University Ningbo China

**Keywords:** antioxidant activity, ascorbic acid, mung bean sprouts, sucrose, total phenolic content

## Abstract

Mung bean seeds were soaked in 0.5 g/L of sucrose solution for 24 hr at 25°C and sprayed with this solution every 12 hr during the germination for 5 days. Our results showed that exogenous sucrose significantly increased vitamin C content throughout germination, and sucrose‐treated sprouts had 23% more vitamin C (20.8 mg/100 g FW) than in control sprouts on day 5. This may be related to higher levels of glucose and l‐galactono‐1, 4‐lactone dehydrogenase activity seen in the treated group versus the control. Total phenolic content and activities of superoxide dismutase, catalase, and ascorbate peroxidase were significantly higher in sucrose‐treated mung bean sprouts than the controls, which contributed to the higher antioxidant activity in sucrose‐treated sprouts. These results indicate that exogenous sucrose treatment increases the content of vitamin C and total phenolics, and enhances the antioxidant activity in mung bean sprouts. It suggests that exogenous sucrose treatment could be an effective technique for producing mung bean sprouts with more vitamin C and higher antioxidant capacity.

## Graphical abstract

Exogenous sucrose increases glucose content in germinating mung bean sprouts. Exogenous sucrose increases vitamin C and total phenolics in mung bean sprouts. Exogenous sucrose enhances total antioxidant activity in mung bean sprouts.

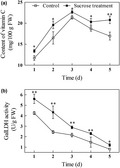

## 1 INTRODUCTION

Mung bean (*Vigna radiata*) sprouts are one of the most common vegetables consumed in China and many other Asian countries; they are also used as a fresh salad vegetable in western countries (Ebert, Chang, Yan, & Yang, 2017). Compared to the seeds, mung bean sprouts contain significantly higher amounts of vitamin C, phenolics, flavonoids, organic acids, and amino acids, and total antioxidant activity (Guo, Li, Tang, & Liu, 2012; Sikora & Świeca, 2018). Tang, Dong, Ren, Li, and He (2014) have suggested that mung bean sprouts contain important functional ingredients for the human diet because of their multifarious biological activities. Recent research has shown that the nutritional and functional quality of mung bean sprouts can be enhanced by treatments such as exogenous ethylene (Liu, Cao, Huang, Guo, & Kang, 2013), ethanol vapors (Goyal, Siddiqui, Upadhyay, & Soni, 2014), oxalic acid spraying (Jin et al., 2016), NaCl‐CaCl_2_ (Yan, Wang, Zhou, Gu, & Yang, 2017)_,_ and ultraviolet‐B (UV‐B) radiation (Wang et al., 2017). Chen, Zhou, et al. (2018) also reported that exogenous ATP immersing could notably maintain the storage quality of mung bean sprouts.

Soluble sugars, especially sucrose, glucose, and fructose, play a central role in plant structure and metabolism at the cellular and organismal levels (Couée, Sulmon, Gouesbet, & Amrani, 2006). During the germination and sprouting of mung beans, soluble sugars accumulate in mung bean sprouts by activating sugar metabolism, which result in an increase in secondary metabolites such as ascorbic acid and phenolics (Chen, Wu, et al., 2019). Sucrose plays a particularly important role, as it is the major form of translocated sugars in plants; it is also the most frequently used sugar in studies of plant sugar responses in gene regulation and development. Application of exogenous sucrose results in ascorbic acid (also called vitamin C) accumulation in harvested broccoli florets by up‐regulating gene expression related to ascorbic acid metabolism (Nishikawa et al., 2005). Exogenous sucrose also improves the nutritional value and antioxidant activity of broccoli florets (Xu et al., 2016), as well as significantly increasing the content of ascorbic acid, anthocyanins, and polyphenols in broccoli sprouts (Guo, Yuan, & Wang, 2011). Morkunas, Marczak, Stachowiak, and Stobiecki (2005) have shown that exogenous sucrose causes a marked increase in endogenous concentrations of soluble sugars (sucrose, glucose, and fructose), isoflavone glycosides, and free aglycones in the embryo axes of *Lupinus luteus* L.

In our previous report, we investigated the effect of different concentration of sucrose, glucose, and fructose treatment on vitamin C content in mung bean sprouts for processing and found that 0.5 g/L of sucrose solution increased vitamin C without any significant effect on the germination rate or growth of sprouts (Qiu, Liu, Ni, Liu & Shao, 2015). Thus, the main objective of this study was to investigate the effects of exogenous sucrose treatment on the levels of vitamin C, some antioxidants, and antioxidant activities during the germination and development of mung beans.

## 2 MATERIAL AND METHODS

### 2.1 Germination conditions

Dry mung bean (*Vigna radiata*) seeds were purchased from Lulin market in Ningbo, China. The seeds were washed with tap water and then randomly divided into two groups, experimental and control. Seeds were soaked in either 0.5 g/L of sucrose solution (pH 7.0), or water for 24 hr at 25°C. After this incubation, seeds were placed into sterile plastic cups with several small drainage holes and incubated in the dark for 5 days at 25°C. Control and sucrose‐treated seeds were sprayed with water and 0.5 g/L of sucrose solution, respectively, every 12 hr during the 5 days of germination. The concentration of sucrose was selected according to the results of our prior study (Qiu et al., 2015). Each treatment consisted of 100 g of mung bean seeds, and the experiment was done in triplicate.

In each treatment, 20 g of mung bean hypocotyls were collected on days 1, 2, 3, 4, and 5 of germination, and then immediately frozen in liquid nitrogen. All frozen samples were stored at −80°C for further analysis.

### 2.2 Measurement of soluble sugar content

The content of soluble sucrose, glucose, and fructose in mung bean sprouts was determined using the same methods described in Wang et al. (2013), including the same extraction method and high‐performance liquid chromatography conditions. Sucrose, glucose, and fructose were identified by their retention times and quantified according to the standards; the results are expressed as mg/g fresh weight.

### 2.3 Measurement of vitamin C content and L‐galactono‐1, 4‐lactone dehydrogenase (GalLDH, EC 1.3.2.3) activity

Vitamin C was extracted from 1.0 g of mung bean hypocotyl cuttings by grinding with 5 ml of 5% phosphoric acid and then centrifuging for 15 min at 12,000 *g* at 4°C. The vitamin C in the supernatants was measured at 525 nm according to the previous method of Kampfenkel, Vanmontagu, and Inze (1995). Ascorbic acid (Analytical Reagent, Solarbio) was used to construct a standard curve. Vitamin C content is expressed as mg/100 g fresh weight.

To determine GalLDH activity, 2.0 g of hypocotyl cuttings were ground with 5 ml cold extraction buffer (100 mM potassium phosphate buffer of pH 7.4) and then centrifuged 5,000 *g* for 10 min at 4°C. Supernatants were collected and centrifuged 12,000 *g* for 20 min at 4°C. Pellets were collected and resuspended in 2 ml of cold extraction buffer. GalLDH activity in this crude extract was assayed according to the method of Tabata, Oba, Suzuki, and Esaka (2001) with some modification. Briefly, 0.20 ml of crude extract was mixed with 2.0 ml of 1.05 mg/ml cytochrome C and then preincubated for 1 min at 25°C. The addition of 0.2 ml of 56 mM l‐galactono‐1, 4‐lactone (GalL) was *t* = 0 of the reaction. GalLDH activity was determined by measuring the change in absorbance at 550 nm. One unit of activity is defined as the amount of enzyme required to oxidize 1 nmol of GalL (equivalent to the formation of 2 nmol of reduced Cytochrome C) per minute.

### 2.4 Measurement of total phenolic content

Approximately 1.0 g of mung bean hypocotyl was ground with 5 ml of 80% ethanol and then centrifuged at 12,000 *g* for 15 min at 4°C. Total phenolic content in the supernatants was measured using Folin–Ciocalteu reagent according to the method of Wang et al. (2017). Gallic acid was used to construct a standard curve, and total phenolic content was expressed as milligrams gallic acid equivalent per gram of fresh weight (mg GAE/g FW).

### 2.5 Measurement of antioxidant enzyme activities

Approximately 1.0 g of hypocotyl was ground with 5 ml of cold extraction buffer (3% polyvinylpyrrolidone, 50 mM potassium phosphate buffer of pH7.0). The sample was centrifuged at 12,000 *g* for 15 min at 4°C; supernatants were collected and used for superoxide dismutase (SOD, http://www.chem.qmul.ac.uk/iubmb/enzyme/EC1/15/1/1.html), catalase (CAT, http://www.chem.qmul.ac.uk/iubmb/enzyme/EC1/11/1/6.html), and ascorbate peroxidase (APX, http://www.chem.qmul.ac.uk/iubmb/enzyme/EC1/11/1/11.html) assays.

SOD activity was determined using the SOD assay kit (Nanjing Jiancheng Institute of Bioengineering, China) according to the manufacturer's instructions. CAT and APX activities were determined as described by Shao, Wang, Xu, and Cheng (2013).

### 2.6 Measurement of total antioxidant activity

Approximately 1.0 g of mung bean hypocotyl was ground with 5 ml of 80% ethanol and then centrifuged at 12,000 *g* for 15 min at 4°C. Supernatants were collected, and the total antioxidant activity was determined by the FRAP (ferric reducing antioxidant power) assay and the DPPH (1,1‐diphenyl‐2‐picrylhydrazyl) radical scavenging capacity assay. FRAP was determined using the total antioxidant capacity assay kit (FRAP method), purchased from Nanjing Jiancheng Institute of Bioengineering, according to the manufacturer's instructions. DPPH radical scavenging was determined by the method of Xu et al. (2016). Briefly, 2 ml of 0.2 mM DPPH ethyl alcohol and 2 ml of extract were mixed well and incubated in the dark for 30 min at room temperature; then, the absorbance at 517 nm was measured. DPPH radical scavenging activity (%) = 100 − (absorbance of sample/absorbance of control) × 100.

### 2.7 Statistical analysis

Statistical analysis was performed using the SPSS package version 13.0 (SPSS Inc.). Data were analyzed by one‐way ANOVA, followed by Duncan's multiple range tests. Values are expressed as mean ± standard deviation (*SD*) in the figures. Differences were considered significant at *p* < .05.

## 3 RESULTS AND DISCUSSION

### 3.1 Effect of exogenous sucrose on soluble sugar content in germinating mung beans

**Figure 1:**
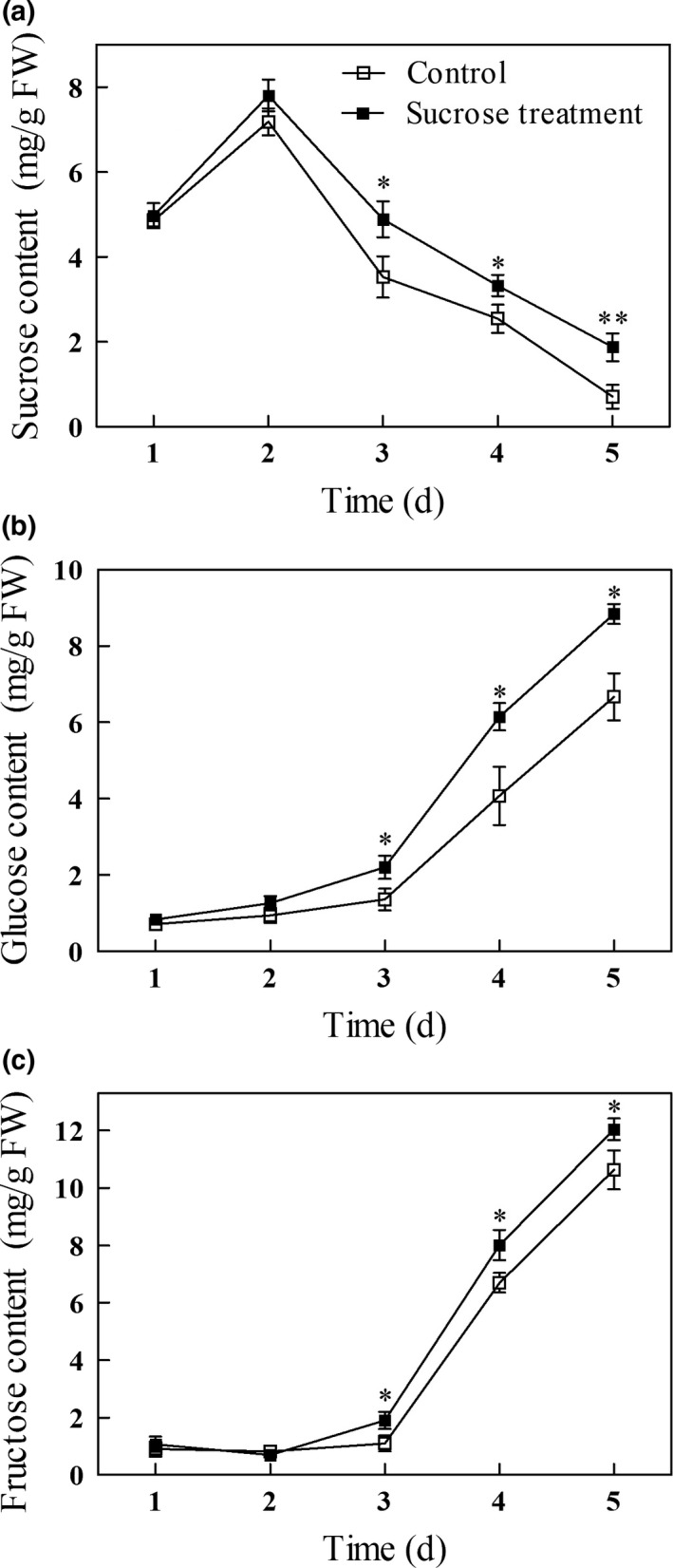
Sucrose (a), glucose (b), and fructose (c) levels in germinating mung bean sprouts. Asterisks indicate significant differences between mung beans treated with exogenous sucrose and the untreated controls (Duncan's multiple range test; ∗, *p* < .05; ∗∗, *p* < .01)

In both sucrose‐treated and untreated mung bean sprouts, the level of sucrose rose sharply between days 1 and 2 of germination and then decreased sharply thereafter (Figure 1a). The levels of glucose and fructose however increased slowly between days 1 and 3, and then increased rapidly thereafter (Figure 1b and c). The increase of endogenous sucrose early in germination may be due to the hydrolysis of oligosaccharides, while the decrease of sucrose content after the 2nd day may be the result of sucrose hydrolysis, which leads to the increase of fructose and glucose. These results are consistent with those reported by (Jom, Frank, & Engel, 2011). In all cases, the levels of soluble sugar in the sucrose‐treated mung beans are significantly higher than in nontreated beans on days 3–5 (*p* < .05); these results are consistent with numerous other reports.

In our previous report, we found that 0.5 g/L of sucrose treatment had the highest vitamin C content in mung bean sprouts among different sugar (sucrose, glucose, and fructose) with different concentration treatments (Qiu et al., 2015). Nishikawa et al. (2005) demonstrated that the content of reducing sugars and sucrose increased significantly in florets when 10% (w/v) sucrose solution was applied to the cut surface of stem tissue in harvested broccoli. Exogenous sucrose also caused a marked increase in concentrations of endogenous sucrose, fructose, and glucose in embryo axes of lupine (Morkunas et al., 2005). Cao et al. (2014) found that pretreatment with 50 mM sucrose leads to higher levels of endogenous sucrose, fructose, and glucose in cucumber seedlings.

### 3.2 Effect of exogenous sucrose on vitamin C levels and GalLDH activity

**Figure 2:**
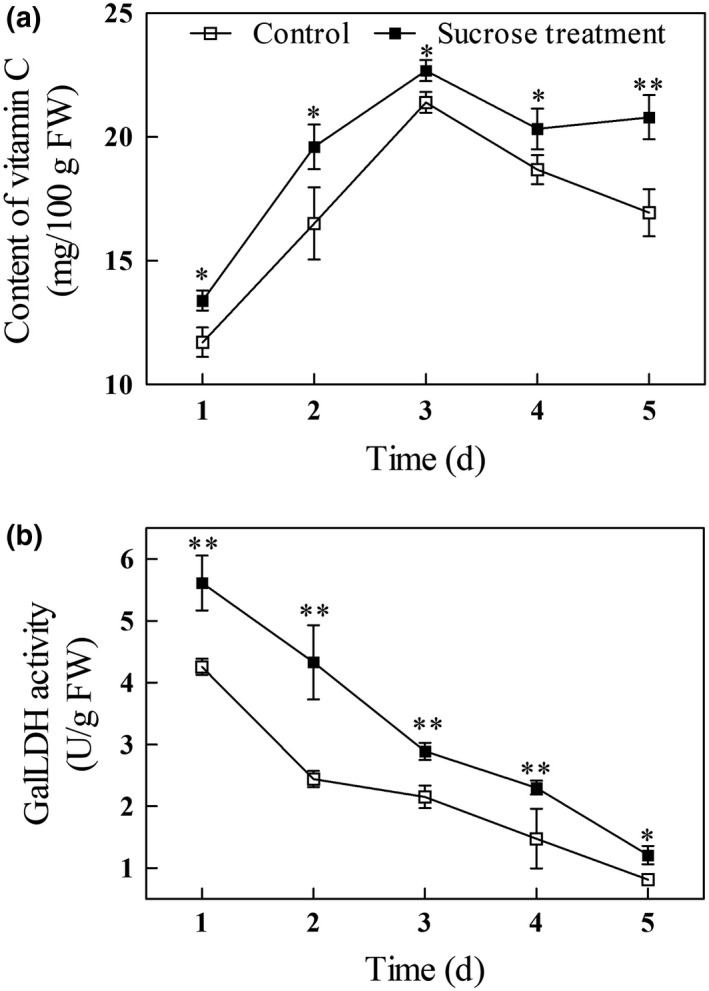
Vitamin C levels (a) and GalLDH activity (b) in germinating mung bean sprouts. Asterisks indicate significant differences between mung beans treated with exogenous sucrose and the untreated controls (Duncan's multiple range test; *, *p* < .05; **, *p* < .01)

As shown in Figure 2a, in both sucrose‐treated and untreated mung bean seeds the level of vitamin C rose sharply between days 1 and 3 of germination, and then declined. At all the time points, there was significantly more vitamin C in the sprouts of the sucrose‐treated seeds (*p* < .05). On day 5 of germination, the treated sprouts had 23% more vitamin C (20.8 mg/100 g FW), than in control sprouts. Vitamin C, also known as ascorbic acid, has strong antioxidant activity and is a cofactor for enzymes catalyzing numerous biochemical reactions, including those neutralizing the effects of reactive oxygen species (Smirnoff, Conklin, & Loewus, 2001; Wheeler, Jones, & Smirnoff, 1998). For a limited number of animals, including humans, that are incapable of its synthesis, vitamin C must be secured by dietary uptake (Giovannoni, 2007). One serving of mung bean sprouts (about 104 g fresh weight) provides 21.6 mg of vitamin C, about 36% of daily required value (Guo et al., 2012).

It has been reported that application of 88 and 176 mM sucrose increased the content of vitamin C in broccoli sprouts by 41% and 26%, respectively, compared with untreated controls (Guo et al., 2011). Cao et al. (2015) also found that sucrose pretreatment increased the content of ascorbic acid in cucumber seedlings. Nishikawa et al. (2005) reported that exogenous sucrose not only resulted in the increase of soluble sugars, but also suppressed the decline of vitamin C. In plant, the ascorbic acid biosynthetic pathway involves a number of steps that converts D‐glucose to ascorbic acid; the last step, where ascorbic acid is synthesized from GalL, is catalyzed by GalLDH (Wheeler et al., 1998). Wei, Xu, and Shao (2017) showed that glucose levels correlated positively with ascorbic acid levels in loquat fruit. GalLDH transcript levels were positively correlated with ascorbic acid levels in carrot (Wang et al., 2015) and peach (Wang, Shao, Gong, Xu & Wang, 2014) fruits. Wang et al. (2017) also reported that GalLDH activity positively correlated with vitamin C levels in mung bean sprouts.

We found that GalLDH activity declined during germination in both the control and treated groups, although the sprouts from sucrose‐treated seeds had significantly higher GalLDH activity throughout germination than those in the control (Figure 2b). This result is similar to that reported by Nishikawa et al. (2005), who showed increased of transcription of *GalLDH* and the suppression of vitamin C loss in sucrose‐treated broccoli florets. Ogawa, Fujita, and Toyofuku (2014) demonstrated that increased glucose levels and GalLDH activity in lettuce and spinach resulted in an increase in ascorbic acid content. We conclude that higher glucose content and GalLDH activity result in increased levels of vitamin C in sprouts of sucrose‐treated mung bean seeds.

### 3.3 Effect of exogenous sucrose on total phenolic content in germinating mung beans

**Figure 3:**
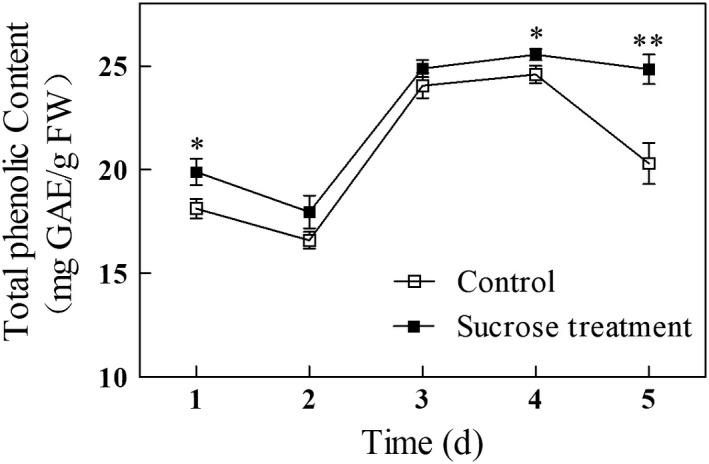
Total phenolic content in mung bean sprouts during germination. Asterisks indicate significant differences between mung beans treated with exogenous sucrose and the untreated controls (Duncan's multiple range test; *, *p* < .05; **, *p* < .01)

Phenolics are the products of secondary metabolism in plants and are thought to provide health benefits associated with reduced risk of chronic diseases (Guo et al., 2012). Here, we found that the total phenolic content of mung bean sprouts from treated and control seeds decreased slightly between days 1 and 2 of germination, then increased sharply to day 3 and then leveled off by day 4. Between days 4 and 5, the total phenolic content in sprouts forms the sucrose‐treated seeds remained high while in sprouts from control seeds, total phenolic content decreased sharply. The difference in total phenolic content between treated and control on day 5 was 22% (Figure 3). Liu et al. (2013) found ethephon treatment increased total phenols in mung bean sprouts by 31% by the second day of germination, but there was no significant difference between control and ethephon treated sprouts at day 4 or 5. Yan et al. (2017) reported no significant difference in total phenolic content in mung bean sprouts from NaCl‐CaCl_2_ treated seeds and control. UV‐B treatment enhanced total phenolic content in mung bean sprouts by 2.9% by the end of germination (Wang et al., 2017). Compared with these results, exogenous sucrose treatment is an effective method for increasing the total phenolic content in mung bean sprouts.

### 3.4 Effect of exogenous sucrose on the activities of antioxidant enzymes in germinating mung beans

**Figure 4:**
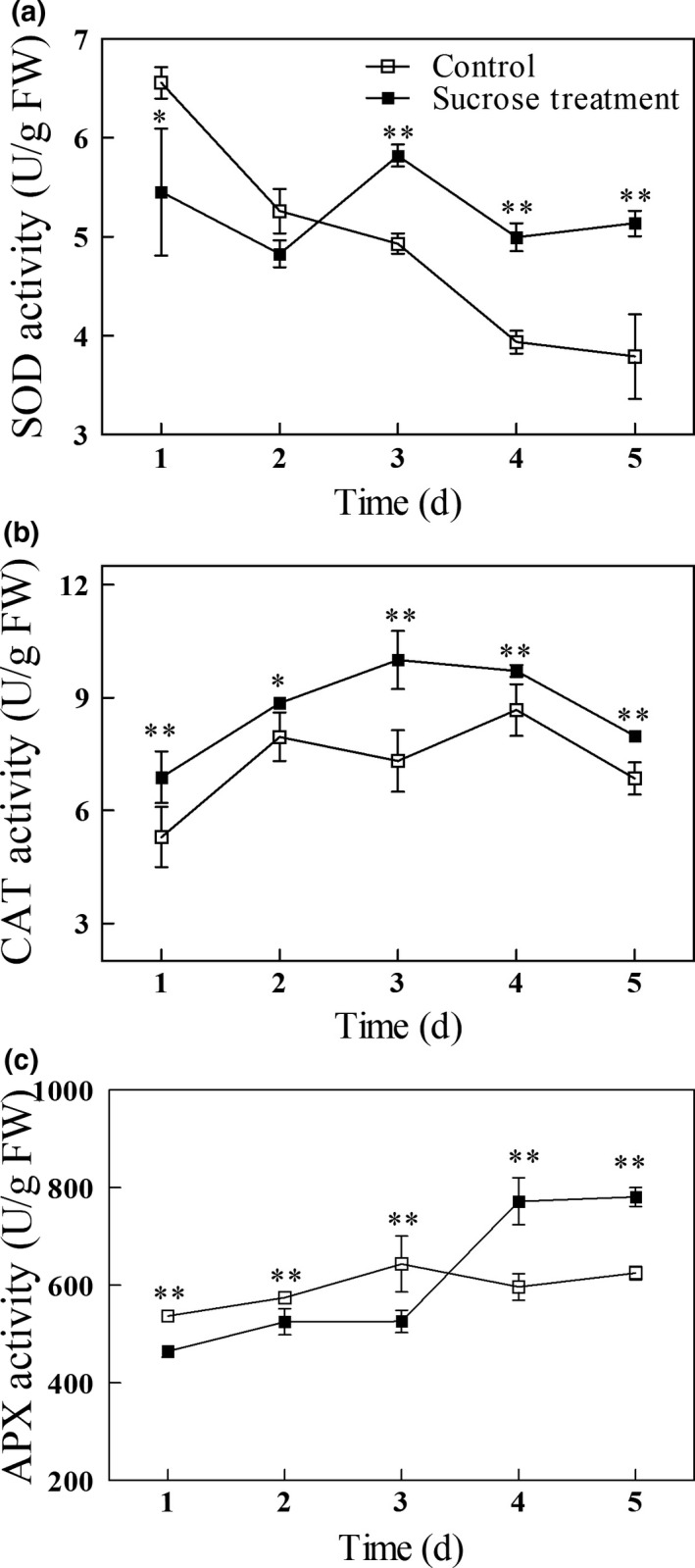
SOD (a), CAT (b), and APX (c) activity in germinating mung bean sprouts. Asterisks indicate significant differences between mung beans treated with exogenous sucrose and the untreated controls (Duncan's multiple range test; *, *p* < .05; **, *p* < .01)

The antioxidant enzymes SOD, CAT, and APX were examined (Figure 4). In both sucrose‐treated and the control groups, SOD activity declined between the first day and second day of germination. In the control group, SOD activity continued declining through day 5, while in the sucrose‐treated group activity increased again and remained significantly (*p* < .01) higher than the controls through day 5 (Figure 4a). CAT activity in both groups had the same general pattern, increasing at first then declining later in germination. Sprouts in the sucrose‐treated group had significantly (*p* < .05) higher CAT activity throughout germination (Figure 4b). APX activity in control and treated sprouts increased slightly between days 1 and 3 of germination then leveled off in control sprouts while increasing sharply in treated sprouts between days 3 and 4 and leveling off between days 4 and 5. Until day 3 of germination, APX activity in the treated sprouts was significantly (*p* < .01) lower than in controls, while on days 4 and 5 it was significantly (*p* < .01) higher (Figure 4c). In 5‐day‐old sprouts, SOD, CAT, and APX activities in the treated group were higher by 35%, 17%, and 25%, respectively, than in controls.

Our results are in agreement with those reported by Cao et al. (2014), who found that in cucumber seedlings exogenous sucrose treatment resulted in higher endogenous sucrose, which thereby activated SOD and APX. The activity of SOD, APX, and CAT in sucrose‐treated florets was also higher than those in untreated controls (Xu et al., 2016).

SOD removes O_2_ **^‐^** by catalyzing its dismutation into either O_2_ or H_2_O_2_. CAT catalyzes the reaction of H_2_O_2_ into H_2_O and O_2_ (Jin et al., 2017). APX catalyzes the H_2_O_2_‐dependent oxidation ascorbic acid with high specificity. Because of the increased GalLDH activity in sucrose‐treated spouts (Figure 2b), we suggest that the lower APX activities (Figure 4c) may result in accumulation of vitamin C (Figure 2a) early in germination, while the higher APX activity at day 4 and day 5 may result in stable levels of vitamin C.

### 3.5 Effect of exogenous sucrose on antioxidant activity in germinating mung beans

**Figure 5:**
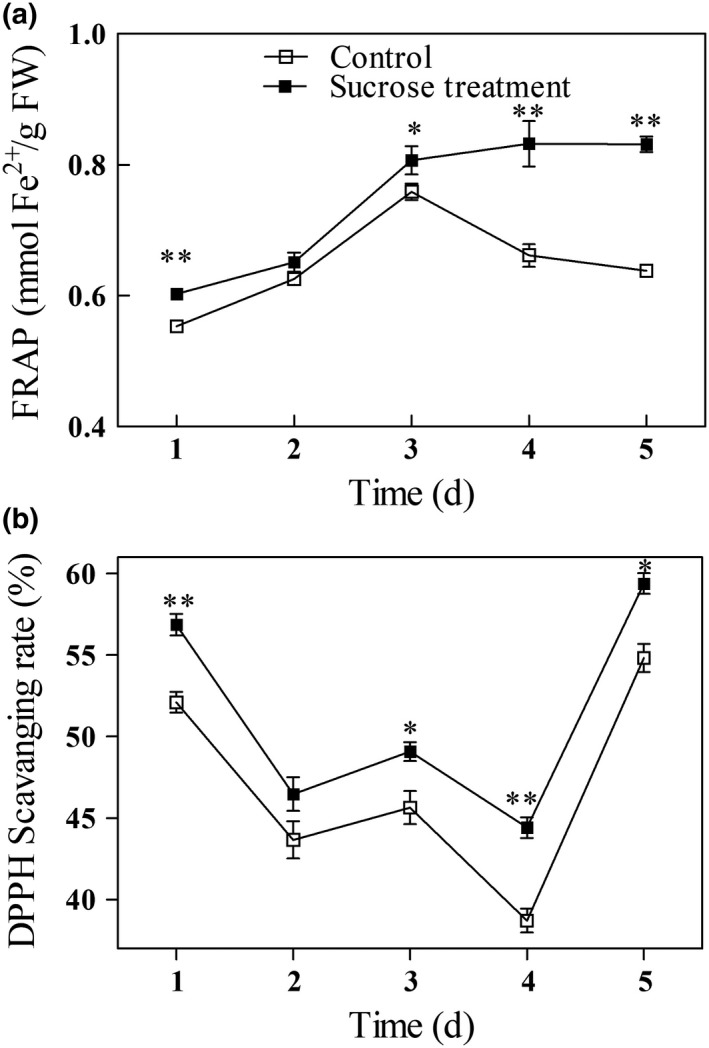
FRAP (a) and DPPH scavenging rate (b) in germinating mung bean sprouts. Asterisks indicate significant differences between mung beans treated with exogenous sucrose and the untreated controls (Duncan's multiple range test; *, *p* < .05; **, *p* < .01)

The antioxidant activities of mung bean sprouts were exhibited by FRAP (Figure 5a) and DPPH scavenging rate (Figure 5b). In both groups, FRAP activity increased to about the same levels between days 1 and 3 of germination. On days 4 and 5, FRAP activity in the control sprouts dropped from the day 3 high of 0.76 mmol Fe^2+^/g FW to 0.66 and 0.64 mmol Fe^2+^/g FW, respectively, while in sprouts from treated seeds, FRAP levels increased slightly to 0.83 mmol Fe^2+^/g FW. The DPPH scavenging rate in both groups declined to the lowest value at day 4 and then increased sharply to the highest levels at day 5. The DPPH scavenging rate in sprouts from treated seeds was consistently higher than the rate in the control sprouts during the germination; 15% and 8.4% higher on days 4 and 5, respectively.

Liu et al. (2013) have demonstrated that increasing the antioxidant activity in mung bean sprouts increases their nutritional value. Jin et al. (2016) suggested that higher antioxidant activities in oxalic acid sprayed mung bean sprouts are due to their higher total phenolic content. The higher DPPH radical scavenging activity in sucrose‐treated broccoli is consistent with their higher levels of total phenols (Xu et al., 2016). In this study, we found that the changes in phenolic content did not exactly coincided with the changes in antioxidant activity during the germination period; however, levels of vitamin C, total phenolics, and the activities of SOD, CAT, and APX all contribute to the higher total antioxidant activity.

### 3.6 The relationship between soluble sugar content, vitamin C content, antioxidant enzyme activities, and antioxidant capacity in mung bean sprouts

During mung bean seed germination and development, antioxidant properties increase markedly by activating the antioxidant enzyme system and accumulating nonenzymatic antioxidants, such as phenolics and other bioactive compounds (Chen, Tan, Zhao, Yang & Yang, 2019). As shown in Table 1, FRAP in mung bean sprouts was positively correlated with levels of reducing sugar, vitamin C, and total phenolics. A significant correlation was observed between the levels of FRAP, and activities of CAT and APX. Because of the high level of total phenolics in mung bean sprouts, the benefits of DPPH radical scavenging are evident (Tang et al., 2014). DPPH and FRAP methods are often used to measure antioxidant capacities. Chen, Tan, et al. (2018) found that there was significant positive correlation between total phenolics content and antioxidant activities tested by DPPH and FRAP assays during the germination and sprouting of broccoli. In this work, we found that exogenous sucrose treatment increased total phenolic content and enhanced antioxidant activities determined by DPPH and FRAP methods during sprouting of mung bean, only FRAP value and total phenolic content were highly correlated (*r* = 0.819, *p* ≤ .01). In the germination and development of mung bean sprouts from seeds, levels of some other ingredients with free radical scavenging activity also change, such as vitamin C, vitamin E, and flavonoids, which would influence DPPH scavenging rate. We also found that glucose levels were positively correlated with levels of vitamin C and total phenolics content. Therefore, exogenous sucrose treatment significantly improved the antioxidant capacity of mung bean sprouts related to the higher levels of reducing sugar, vitamin C, and total phenolic, as well as antioxidant enzymes activities.

**Table 1:** Correlations between the content of soluble sugar, vitamin C, the activity of SOD, CAT, and APX, and total antioxidant capacity in germinating mung bean sprouts

	Sucrose content	Fructose content	Glucose content	Vitamin C content	GalLDH activity	SOD activity	CAT activity	APX activity	Total phenolic content
Vitamin C content	−0.140	0.268	0.367*						
Total phenolic content	0.505**	0.552**	0.552**						
FRAP	−0.317	0.557**	0.557**	0.856**	−0.463**	−0.071	0.687**	0.701**	0.819**
DPPH	0.289	0.297	0.297	−0.244	0.156	0.276	−0.437*	0.031	−0.078

* Indicates significant correlation at *p* ≤ .05.

** Indicates significant highly correlation at *p* ≤ .01.

## 4 CONCLUSION

Mung bean sprouts are an excellent source of bioactive compounds with high antioxidant activity. Treatment of mung bean seeds with 0.5 g/L of sucrose resulted in mung bean sprouts with increased levels of vitamin C, total phenolics, SOD, CAT, and APX activities, and total antioxidant activity, over untreated controls. At the end of sprouting (day 5), sucrose‐treated mung bean sprouts showed 23% more vitamin C (20.8 mg/100g FW) and 22% higher total phenolic content (24.8 mg GAE/g FW) than the control sprouts. Thus, exogenous sucrose treatment could be an effective technique for producing mung bean sprouts with high antioxidant capacity.

## CONFLICT OF INTEREST

The authors have declared no conflicts of interest for this article.

## ETHICAL APPROVAL

This study does not involve any human or animal testing.

## INFORMED CONSENT

Written informed consent was obtained from all study participants.
